# *‘Community people are the most powerful resources’*: qualitative critical realist analysis and framework to support co-produced responses to zoonotic disease threats with(in) Nepali communities

**DOI:** 10.1186/s12889-025-22657-9

**Published:** 2025-04-16

**Authors:** Anna Durrance-Bagale, Hari Basnet, Nanda Bahadur Singh, Steven R. Belmain, James W. Rudge, Natasha Howard

**Affiliations:** 1 London School of Hygiene & Tropical Medicine, Department of Global Health & Development, 15-17 Tavistock Place, London, WC1H 9SH UK; 2 Nepalese Ornithological Union, Kathmandu, Nepal; 3 Central Department of Zoology, Tribhuvan University, Kathmandu, Nepal; 4 Natural Resources Institute, University of Greenwich, Chatham Maritime, Kent, ME4 4TB UK; 5 Faculty of Public Health, Mahidol University, 420/1 Rajvithi Road, Bangkok, Thailand; 6 Saw Swee Hock School of Public Health, National University of Singapore, National University Health System, 12 Science Drive 2, Singapore, Singapore

**Keywords:** Co-production, Community engagement, Infectious disease, Mitigation, Nepal, One health, Zoonotic disease

## Abstract

**Background:**

Co-production between researchers, service providers, and members of affected communities is an old concept renewed by current efforts to decolonise global health, reduce exploitative practices, and develop more sustainable, context-relevant interventions to address global health issues. Working with communities– how ever defined– is central to healthcare improvement but engaging with communities and identifying priorities remains challenging for disease control professionals. Co-production aims to help ensure community members have some control over the design and implementation of any intervention, and greater ownership of processes and outcomes. We aimed to identify what would encourage co-production of activities to prevent potential transmission of zoonoses.

**Methods:**

In this qualitative study, we (British and Nepali researchers) interviewed 73 participants from six communities across Nepal, with 10 participating in photovoice. We also interviewed 20 healthcare professionals and policymakers, 14 representing human and six representing animal health. We interpreted data using reflexive thematic analysis.

**Results:**

Thirty-nine people in six communities participated in interviews, with another 34 in 5 focus groups. We generated three overarching themes: (i) constrained healthcare-seeking behaviours, (ii) experience of community programmes, and (iii) community priorities and co-production. Community participants, despite strong opinions and desire to participate in disease control interventions, had experienced little or no attempt by intervention organisers to engage them in design, implementation, evaluation, or accountability. Most had no experience of programmes at all. Participants highlighted the significance of working in ‘local’ languages, respecting religious and cultural realities, relating initiatives to lived experience, and ensuring that local leaders are involved.

**Conclusions:**

Meaningful co-production requires recognising communities– through legitimate leadership/representation– as expert and equal partners who can ‘work alongside’ at all stages of any initiative. Implications from this research include the importance of promoting trust in communities through inclusion of influential community members (community health volunteers, traditional medicine practitioners, women’s group leaders); the use of indigenous languages; the acceptability of different media for interventions (theatre, drama); and the need to be pragmatic about available resources, to manage the expectations of community members.

## Background

Zoonotic diseases are a rapidly growing threat, epitomised by impacts of the COVID-19 pandemic on the health and economic status of populations globally [1]. With over 60% of infectious diseases in humans caused by pathogens shared with domestic or wild animals [2, 3], sustainable, One Health approaches to communicable disease management are paramount. One Health has recently been defined as ‘an integrated, unifying approach that aims to sustainably balance and optimize the health of people, animals, and ecosystems’ [4]. Zoonoses are a particular threat in under-resourced countries such as Nepal where much of the population, especially in rural areas, may have little access to functioning health facilities [5], and depend on livestock-keeping for their livelihood, necessitating close human-animal contact that could present disease risks [6, 7]. As a lower-middle income country with an annual spend of around US$53 per capita on healthcare [8, 9] there is little scope for Nepal to develop advanced surveillance systems to identify potential infectious disease threats, and there is little research detailing burden from these diseases in the country.

Finding cost-effective ways to ensure that healthcare initiatives work is important, especially in resource-poor countries, where the most vulnerable people may not have the capacity– financial or otherwise– to implement interventions, and where, if sufficiently engaged, they themselves can play a role in delivery of health outcomes [10, 11]. Putting people at the centre of any programme initiation or implementation, asking them about their priorities, is crucial, as is considering issues such as cultural, social and religious norms and beliefs, ethics, inclusivity, and power relations, which may affect both a community’s willingness to discuss and participate, and also the success of a programme [10, 12–15]. Initiatives are more likely to work if communities have been actively involved in planning solutions so that they are culturally and context-sensitive (what works ‘here’ may not work ‘there’) and been given the chance to discuss their actual situation rather than how this is perceived by outsiders [11, 15–17]. Feasibility and recognition of what assets are available is central here: there is little point recommending an expensive or time-consuming ‘solution’ to an issue if people can afford neither the time nor the expense of these potential solutions [11, 15–17]. Community members’ perceptions of what is likely to work may be more realistic as they are working with what they have, rather than an ideal-world scenario. This was demonstrated clearly during the Ebola Virus Disease epidemic in west Africa in 2013–2016, where the response was, to an extent, shaped by the communities affected [18, 19]. Taking into account underserved populations, e.g., community members who have low literacy, or those living in informal settlements with limited access to healthcare and frequent contact with synanthropic rodents or community dogs [20], is also important, to try and reach as many people as possible, and particularly those who might be most affected by threats to their health. For the purposes of this research, we defined a community as ‘residents of settlements where health research is conducted, potential study participants, all other residents in the immediate locality’ [17].

Community engagement, here defined as ‘the process of working collaboratively with and through groups of people affiliated by geographic proximity, special interest, or similar situations to address issues affecting the well-being of those people’ has a two-fold purpose in healthcare provision: (i) to improve the health of individuals and communities; and (ii) to empower people, helping them achieve some control over this facet of their life [21, 22]. Co-production in healthcare aims to ensure that communities have some control over the design and implementation of any intervention, gives them ownership of the process and the outcome, and should allow them to hold health providers to account [13, 23]. The main aims of community engagement are to enable communities to ‘define their own needs and solutions’, while supporting them to do so, to make services more responsive and effective [24], while that of co-production is to ensure that potential or actual end-users (e.g., members of a particular community) are able to work together with service providers (e.g., healthcare professionals, policymakers, academics) to produce knowledge and interventions that are useful, workable, and (cost-)effective in the context in which they are used. Co-production, in the context of zoonotic disease specifically, has received relatively little attention, despite the fact that it has been demonstrated to allow identification of disease spillover routes, and can help inform disease control strategies [6]. A meta-ethnography of participatory health research and co-production in Nepal suggests that, although this approach is becoming more common in the country, it is important to ensure that research and implementation is appropriate to the participants, allowing them input into design, and addressing issues and mitigation that they suggest are most relevant [25].

Lack of evidence on risk factors and drivers that increase potential for disease emergence in communities impedes the design of appropriate mitigatory strategies [26]. Indigenous knowledge and practices are likely to be an untapped source of information and people may already take preventative actions, even if this is not clearly articulated. Active participation may increase trust and help ensure that solutions are relevant and context-sensitive [27]. In a recent scoping review on anthropogenic factors that may increase zoonotic disease risk in the Indian subcontinent, we concluded that simply promoting community knowledge and awareness will not result in behaviour change, and that working with and in communities, co-designing both research and implementation, is key to successful, relevant and context-specific interventions [28].

Fundamental Cause Theory, described by Link and Phelan in 1995, states the importance of contextualisation of identified risk factors for illness, with a focus on social factors such as socio-economic status and social support (e.g., access to resources) as ‘fundamental causes’ of disease [29, 30]. These fundamental causes are important because they are part of a wider constellation of factors that drive mechanisms that cause illness, although they are not the only causal mechanisms. Link and Phelan argue that individual factors should be contextualised to identify why people are at risk of disease. If we do not do this, we ignore the many dynamic processes that work on these factors to produce disease risk [29, 30]. This theory, while important and useful, focuses primarily on the contextualisation of social and socio-economic factors, rather than more subtle, implicit, harder-to-explain factors such as individual perceptions, power relations, and religious and cultural beliefs [31]. Community and individual priorities may well be different to those anticipated by researchers or healthcare professionals. Communities may be accustomed to living with a disease and not perceive it as a priority or reason to seek treatment, or have different explanations for illness [32]. This highlights the central importance of working with communities rather than implementing practices or policies that may be irrelevant to the community in question.

We thus aimed to identify what would encourage community co-production of activities to prevent potential transmission of zoonoses, through discussions in geographically situated communities and interviews with Nepali stakeholders working in human, animal or environmental health. Research on this issue is lacking in Nepal, particularly qualitative work focusing on the views and opinions of participants during the development and implementation of interventions. Therefore, the first and second author, with input from colleagues in Nepal on study design and analysis, initiated this exploratory study. Rather than co-producing a piece of research, we first had to identify, through discussion with people in the communities and sectors involved, what might make this process effective in future.

## Methods

### Study design

We conducted a multimethod, qualitative study, incorporating interviews, photovoice, focus group discussions (FGDs) and unstructured observations with community members, and interviews with health-workers, veterinarians, and policymakers.

The study was informed by critical realism: a philosophy of science combining a realist ontology (the world exists independently of us) and subjective epistemology (we can observe the world and draw conclusions, but these are fallible), in which an understanding of the specific context in which people live and work is central [33–35]. We held a participatory axiology, which recognises the importance of community members and their views and experience in addressing issues that affect them: here, risk of zoonotic disease. This axiology can inform policy and programming, e.g., co-production or co-design of research and interventions, as it recognises that marginalising outcomes can result from individual and systemic factors [33, 34, 36].

### Study sites

**Fig. 1:**
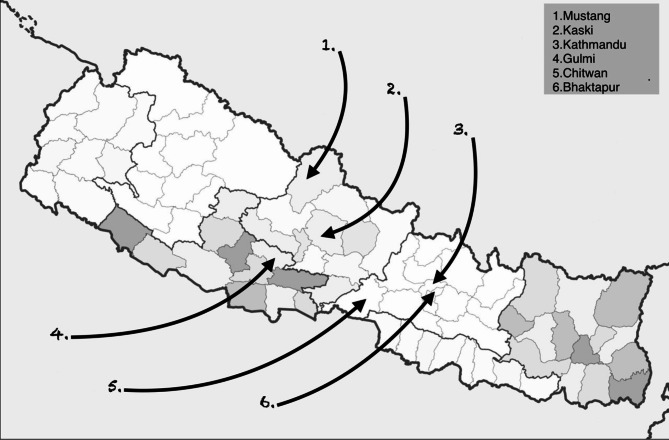
Map of sites

We selected six sites after discussion with Nepali colleagues, including one informal settlement in the Kathmandu valley (Fig. 1). Three sites– Kaski (Pokhara; second-largest city in Nepal), Kathmandu (national capital city) and Bhaktapur– are largely urban, while Mustang and Gulmi are rural and remote. Chitwan is situated in the lowland (Terai) and is a national park where many people earn a living from animal-focused tourism, while Mustang is mountainous and sparsely populated. Most participants identified as Hindu, while others stated that they were Buddhist, particularly in Mustang. All participants spoke fluent Nepali. Many residents of Bhaktapur identified as Newar, a distinct group with its own language, Newari, as well as Nepali.

### Participant sampling and recruitment

The study enrolled both community members and healthcare professionals/policymakers working in human, animal or environmental health, to gain knowledge on how both sets of participants define and perceive the pertinent issues. We used a combination of typical sampling and snowballing [37, 38]. At each site, we aimed to enrol six to eight adult participants for individual interviews and six to 10 participants for one FGD. This sample size was judged sufficient to develop an understanding of the views, opinions and experiences of participants, but was open to adjustment (e.g., if we were not collecting informative data) [38]. We contacted a local healthcare worker (if available) and asked them to suggest potential participants. If a healthcare worker was not available, we identified a prominent community member, for example a member of a community group (e.g., a local women’s group) or a teacher, and asked them to suggest participants. ADB and HB met potential participants to explain the aims of the study, and ran interviews if they were willing to participate. We asked each participant to identify another potential participant until we had recruited approximately six to eight individuals and one group per community.

ADB generated a seed list of Nepali healthcare professionals and national or regional policymakers, and asked Nepali colleagues to nominate potential interviewees from relevant organisations. All were contacted by email: two refused due to time constraints and three did not respond. Participants were asked to nominate others who were likely to have useful experiences or viewpoints to discuss at the end of their interview.

### Consent procedures

We explained the study to potential participants before any other study procedure took place. Following their agreement, participants signed a consent form or gave verbal consent confirming that they had read and understood the study information sheet. Consent of illiterate participants was witnessed by someone selected by the participant and unrelated to the study team, after explanation of the documents.

### Data collection

ADB and HB collected data between April and July 2022. Participants were interviewed at home, or in their place of work or a local café, as they judged appropriate, and to allow confidentiality and privacy. All recordings were given an alphanumeric code to ensure confidentiality. Completed consent forms were scanned and shredded. Electronic data were password-protected and only accessible to the study team. Data collection methods were appropriate to critical realist analysis, particularly the combination of interviews, FGDs and observations, which allowed some triangulation and deeper understanding of participants’ perspectives rather than relying on one approach from which to draw conclusions [39].

#### Interviews

Topic guides were informed by findings from our previous literature review [28] and understanding of the context.

The topic guide for community participants focused on human-animal contact, biosecurity and food hygiene, environmental changes, health issues and disease awareness, and experience with awareness programmes. The policymaker and practitioner topic guide covered relevant experience, views on community awareness, and governmental policy on zoonotic and infectious diseases. Additional topics raised by participants were discussed as appropriate during interviews.

Interviews were approximately 30 min and were conducted in Nepali (by HB and ADB) or English (by ADB) as decided by the participant. ADB conducted seven healthcare professional and policymaker interviews remotely using Zoom software (Zoom Video Communications Inc, San Jose) and audio-recorded with automatic transcription enabled. ADB reviewed all transcripts against recordings.

#### FGDs

The FGD guide covered the same topics as individual interviews, lasted around 45–60 min, and were conducted in Nepali. One FGD was conducted in both Nepali and Newari at participants’ request as all participants were fluent in both languages. We included FGDs because they enabled a different type of engagement and more social perspectives than did individual interviews.

#### Unstructured observations

During data collection (interviews, FGDs and photovoice) ADB made brief written observations on the ‘three Cs’ [40]: context, content or concept of potential significance or interest, e.g., if participants laughed when asked about certain topics, or how participants interacted with animals. These observations promoted understanding of relevant contextual factors and how these may relate to discussion topics or behaviours [40].

### Analysis

Recordings in Nepali were transcribed into English by a Nepali public health professional who was fluent in both languages. Two of the first transcriptions were back-translated and reviewed by another native Nepali speaker to ensure that the transcription accurately reflected the recording. Interviews in English were transcribed by ADB. Names used by participants for animal diseases were cross-checked with a Nepali veterinarian fluent in English to ensure that they represented the correct disease.

Interview and FGD transcripts were imported into NVivo software (QSR International Pty Ltd, Version 12, 2018) for data management. Unstructured observation notes were imported into NVivo and used to contextualise events and behaviour and aid in their interpretation [41].

ADB reviewed and coded all transcripts and observations in NVivo, using reflexive thematic analysis to generate themes and sub-themes from the data [42, 43]. The analysis was influenced by the work of Fryer and Wiltshire [44, 45], and involved five steps to allow development of potential causal explanations for phenomena: (i) developing a research question; (ii) becoming familiar with the data; (iii) apply, developing and reviewing codes; (iv) developing and reviewing themes (from codes); and (v) generating findings [45]. While reviewing (step iv) we reflected on and questioned the validity of themes and potential causal explanations, to examine whether they were appropriate and cogent [44].

Critical realism has become a popular theoretical positioning for reflexive thematic analysis [42] and recognises the centrality of the researcher in the interpretation process. Approaching the data from a critical realist stance meant triangulating data from interviews, FGDs and observations, to attempt to understand the reality of participants’ lived experience, extrapolating from their words. This allowed the development of a clear narrative and analysis of the causal mechanisms and contextual factors that may have influenced participants’ perceptions and understandings.

### Reflexivity

Most participants had never been asked to discuss their views and experiences on any issue by anyone, particularly a researcher from a western country. While at times disadvantageous, outsider status can also be an advantage, as the personal experiences of ‘insiders’– people perceived as part of the community– may affect what they discuss with participants, and how they relate to each other [46]. Despite having a relatively good understanding of Nepali culture, and having lived and worked in Nepal intermittently since 2006, ADB, a white British female researcher, was aware that she brought herself into initiating, planning, conducting, and analysing the research. Being an objective observer is impossible, as researchers, with their social, political and educational values, their (implicit and explicit) motivations and hopes, are a central part of the research [47–49]. Researchers must remain aware of their ‘foreign gaze’ [50], keeping positionality in mind. HB, a Nepali man, was also aware of how his presence may have affected participants, and discussed any perceived issues with ADB after each interview.

## Results

### Participant characteristics

Thirty-nine people (21 men, 18 women) from the six sites participated in semi-structured interviews and an additional 34 people (14 men, 20 women) participated in five FGDs (Table 1).

**Table 1:** Community participant characteristics

Identifier	Gender	Estimated age	Language
Bhaktapur1	Female	45–50	Nepali
Bhaktapur2	Female	50–55	Nepali
Bhaktapur3	Male	35–40	Nepali
Bhaktapur4	Female	30–35	English
Chitwan1	Male	45–50	Nepali
Chitwan2	Male	70–75	Nepali
Chitwan3	Female	45–50	Nepali
Chitwan4	Male	40–45	English
Chitwan5	Male	35–40	Nepali
Chitwan6	Male	60–65	Nepali
Gulmi1	Female	65–70	Nepali
Gulmi2	Female	35–40	Nepali
Gulmi3	Female	55–60	Nepali
Gulmi4	Female	45–50	Nepali
Gulmi5	Male	60–65	Nepali
Gulmi6	Female	55–60	Nepali
Kathmandu1	Male	25–30	Nepali
Kathmandu2	Male	45–50	Nepali/English
Kathmandu3	Female	35–40	Nepali
Kathmandu4	Female	35–40	Nepali
Kathmandu5	Male	20–25	English
Mustang1	Female	45–50	Nepali
Mustang2	Female	40–45	Nepali
Mustang3	Male	20–25	Nepali
Mustang4	Male	40–45	Nepali
Mustang5	Female	35–40	Nepali
Mustang6	Male	40–45	Nepali
Mustang7	Male	45–50	Nepali
Pokhara1	Male	45–50	Nepali
Pokhara2	Male	20–25	Nepali
Pokhara3	Male	30–35	Nepali
Pokhara4	Female	50–55	Nepali
Pokhara5	Male	45–50	Nepali
Pokhara6	Female	50–55	Nepali
Pokhara7	Male	55–60	Nepali
Pokhara8	Male	55–60	Nepali
Pokhara9	Female	50–55	Nepali
Pokhara10	Female	35–40	Nepali
Pokhara11	Male	55–60	Nepali
Bhaktapur FGD	5 male, 4 female	20–70	Nepali/Newari
Chitwan FGD	1 male, 3 female	20–70	Nepali
Gulmi FGD	0 male, 9 female	20–70	Nepali
Mustang FGD	5 male, 1 female	20–70	Nepali
Pokhara FGD	3 male, 3 female	20–70	Nepali

Twenty Nepali healthcare professionals and policymakers were interviewed in English (Table 2): 14 representing human health (13 men, one woman) and six representing animal health (three men, three women). Location information is not included for these participants to protect their anonymity.

**Table 2:** Healthcare professional participant characteristics

Identifier	Type	Gender	Interview
Health1	Infectious disease specialist	Male	In-person
Health2	Clinician/NGO	Male	In-person
Health3	Public health specialist	Male	In-person
Health4	Consultant for health NGOs/iNGOs	Male	In-person
Health5	Government (central)/NGO	Female	In-person
Health6	Infectious disease specialist	Male	In-person
Health7	Government (central)/clinician	Male	In-person
Health8	Consultant for health NGOs/iNGOs	Male	In-person
Health9	Government (central)/infectious disease specialist	Male	In-person
Health10	Consultant for health NGOs/iNGOs	Male	Remote
Health11	Infectious disease specialist/academic	Male	Remote
Health12	Public health specialist	Male	Remote
Health13	Consultant for health NGOs/iNGOs	Male	Remote
Health14	Government (regional)/public health specialist	Male	Remote
Livestock1	Government (central)/veterinarian	Male	In-person
Livestock2	Government (central)/veterinarian	Male	In-person
Livestock3	Government (regional)/veterinarian	Female	In-person
Livestock4	Government (regional)/veterinarian	Male	In-person
Livestock5	Government (regional)/veterinarian	Female	Remote
Livestock6	Government (central)/veterinarian	Female	Remote

iNGO: international non-governmental organisation; NGO: non-governmental organisation

### Thematic findings

We generated three themes: i) constrained healthcare-seeking behaviours, ii) experience of community programmes, and iii) community priorities and co-production. We report ‘community’ perspectives, followed by ‘policymaker/practitioner’ perspectives under each theme, as relevant. We found no notable differences in responses by geography, but found considerable similarity between ‘community’ and policymaker/practitioner’ perspectives.

### Constrained healthcare-seeking behaviours

In the communities we visited, accessing healthcare usually meant visiting a local health post for non-serious illness and visiting a hospital in the nearest town for more serious events. Health posts are sited in larger villages and provide immunisation, family planning, and maternal healthcare, with basic preventative healthcare services. There are 25 federal hospitals in the entire country, which provide comprehensive healthcare services, including emergency healthcare. There is one specialist infectious disease hospital in Nepal, based in Kathmandu. These hospitals take more time (sometimes days), money and effort to reach:

‘We will first go to clinic [*health post*] because it is more easy for us and quick rather than going in hospital because in hospital it takes a bit long time for ticket and all the systems[…]we go to hospital if we have to do some more detailed check-ups and test.’ [Bhaktapur4].

‘If it is a mild cough and cold, we take them to the health post nearby. Otherwise, we take them to the Jomsom hospital in case of severe condition.’ [Mustang1].

An FGD participant discussed being admitted to hospital for 15 days after contracting an illness from one of her livestock. After experiencing lung complications in the hospital, she got worse:

‘[*It*] was near to death experience and the doctors too were not completely sure of the animals that caused the wound and the illness. The causing agent is still unknown.’ [Chitwan FGD].

Most participants with livestock or pets stated that they would contact a veterinarian if their animals became ill but again they would try other options first, including waiting to see if the animal’s condition improved, and administering traditional medicine:

‘When we feel the buffalo is sick first few days we wait and see whether it eats[…]If it is a little bit serious then we call the veterinary doctor and he comes and then he finds out some problem, he gives injection or whatever.’ [Chitwan4].

‘If the goat does not pee, we feed the leaf of the eggplant. If the homemade treatment does not work, we take them to the vet.’ [Gulmi2].

Lack of veterinary services in the local area adversely affects animal health, with people not able to easily access affordable services if they do exist. This may affect human health in turn, as without effective treatment disease may transmit from livestock to owner:

‘People ask us to take them to the vet but to get there it takes 3–4 days. Till then the buffaloes may already be healthy or have died due to the sickness. There is no proper service here.’ [Pokhara3].

Vaccination of dogs against rabies was widespread, with reasons including disease prevention and ability to demonstrate vaccination records to others:

‘So many street dogs are there nearby my house[…]They do bite and when we got dog bite, we scared that they might be rabies disease. And then people took them to the hospital[…]For their rabies vaccination.’ [Chitwan4].

‘If it is on the vaccination card then it is okay. Only if they [*veterinarian*] see that something is missing on the card, then they will tell[…]it’s a proper record. If the dog bites somebody then we can show that it’s vaccinated.’ [Pokhara2].

This was also true of pet dogs in the informal settlement, where all pet dogs are vaccinated against rabies, as part of a campaign by veterinarians, for which the owner pays 250 rupees (about US$2), according to Kathmandu3.

### Experience of community programmes

No community and few policymaker/practitioner participants had experience of any community awareness or engagement programmes, although some described general health camps (where medical professionals provide basic health check-ups and health advice), and actions taken in their communities to address issues such as nuisance dogs.

#### Limited community programmes

Dog vaccination programmes, which involve some engagement to encourage communities to attend, are organised by local authorities in some areas:

‘People can complain if any dog creates a mess or dirt. People also collect 100 rupees per year and dogs get vaccinated, which started in Pokhara at first. In our ward a notice has been published recently.’ [Pokhara1].

Dog sterilisation was organised in some communities, although this was usually initiated by non-governmental organisations rather than as a formal activity through governmental channels:

‘They sterilise dogs and also kill them if their number increases. These are mostly conducted by NGOs.’ [Bhaktapur FGD].

‘We have given the dog rabies vaccine when people came to us to make us aware. People also made the dog sterile by conducting an operation. People has come to vaccinate her and also took 20 rupees.’ [Pokhara FGD].

Three informal settlement participants independently mentioned a health promotion initiative run by an NGO in the form of a drama session. Presenting information visually and involving the audience was effective for these participants, and they were able to tell us about information that had been discussed during the session. This is particularly important as people who live in these settlements are likely to have low literacy and fewer financial resources to access healthcare:

‘Sometime people come here and distribute medicine for free and also screening and health awareness programmes get conducted. I don’t know where they come from but people do drama and give medicine without cost[…]it’s really helpful for poor people.’ [Kathmandu2].

Health-related information was disseminated in hospitals and health camps, which were often focused on one specific issue, e.g., diabetes or COVID-19. Again, these are often administered by NGOs rather than government:

‘COVID-related awareness programmes and training on how to be safe and wash hands were also conducted. Specific vaccination programmes on measles or diarrhoea were conducted in this area from time to time by the village development councils. These programmes are mostly conducted by rural municipality.’ [Mustang3].

‘Some organisations, NGO or iNGO, they call the public to talk about the diabetes or [*blood*] pressure or other types of disease. They make a camp, which is free camp. Also some time some dentist, they come and they make a camp, and they check up the public villagers’ teeth.’ [Chitwan4].

Community engagement was discussed at length by policymaker/practitioner participants, who explained why involving communities in any programmes designed to benefit them was so important. Firstly, working with community leaders and local health workers who are known to communities and gaining community trust is key, otherwise initiatives are unlikely to work:

‘If they know these people [*community leaders*] are involved then the trust factor is increased. And when the trust factor is enhanced, people seem to work closely with us.’ [Health10].

‘Female community health volunteers organise a monthly meeting among the women’s groups[…]They talk about health issues, like general health issues mainly, sometimes women’s issues. So regarding zoonotic disease as well, we go through them.’ [Health14].

Secondly, involving end-users in the design of programmes and feeding back results ensures that people feel they are being heard and that their contributions have value:

‘Every year we have a meeting[...]there will be the farmers and people from government[…]farmers will say we are facing this problem[…]based on that information, we prioritise the disease and then we can do research on that topic.’ [Livestock3].

‘The data they collect, they present in Nepali in front of them, what is their health status, what are they lacking, what is the nutrition status, so the village development committee’s leader and female community health volunteer teacher, they gather them and present in front of them.’ [Health11].

Thirdly, receiving feedback from community participation helps in designing effective programmes, and ensuring they are relevant to the specific community:

‘[*Do you get good feedback from community members?*] Yes of course, that is a very important part for us.’ [Health11].

Community members are receptive to attending programmes that they feel will help them:

‘We do have really a good experience regarding community people and if we do data collection they will offer a meal, and sometimes they give us gifts too, like vegetables and fruits.’ [Health12].

#### Lack of community-led programmes

There was general agreement among participants that community-led initiatives in general did not exist, with little co-operation between residents. Most control measures were taken by individuals:

‘We have not done any discussion or measure [*to control rodents*] among the village group. We deal with it individually.’ [Chitwan5].

‘The people who have dogs get them vaccinated themselves. But no one in the community has made any plans or programmes for the stray dogs.’ [Gulmi FGD].

One community discussed rodent control practices, including provision of poison from local government:

‘What they do is provide poison by the people from agriculture centre to kill them and also give tips on when not to use it, such as while cultivating the food or around the food.’ [Mustang FGD].

One exception to the consensus around a lack of community-led initiatives was in Mustang. Mustangi participants discussed a women’s group who initiated fines for villagers who allowed their dogs to run around outside the home, worrying animals, destroying crops, and biting people, with the associated threat of rabies:

‘Fines have to be paid by those who does not follow the rule, up to 100 rupees that increases if they still do not follow the rules. These are looked after by the executive members of mothers’ group. Mothers’ group have developed a fund from fines which goes for social works. The money they collect from the fine system are used for cleanup programmes or buying dustbins.’ [Mustang3].

### Community priorities and co-production

#### What communities want to know

There was little general awareness of zoonotic disease among participants at the six study sites. Participants claimed interest in learning more about zoonotic disease and taking part in health programmes in general, especially if they were tailored to health issues relevant to them:

‘I think it is important and these programmes should be conducted by the government rather than the organisations. As a lot of people suffer from sugar [*diabetes*] and [*blood*] pressure, programmes related to this disease should be conducted[…]this information is very important.’ [Bhaktapur1].

‘I want to know about the new diseases. I have only studied till class 3 but want to be more aware. I also make people aware of the information I know like the luto [*demodicidosis*] I talked about.’ [Chitwan6].

‘This is a rural area and many people are rearing pet animals like dogs, cats, pigs, hens but most of the people don’t know that disease get transmitted from animals inside the home. Such a programme would definitely help people to know about such disease and can prevent themselves [*becoming ill*].’ [Pokhara10].

Only one participant suggested that awareness campaigns were irrelevant for her because neither she nor her animals were ever ill:

‘I don’t care for it [*knowledge on zoonotic disease*]. Our animals and we haven’t been sick in a long time[…]the rangers from forest and sometimes others come and give us some information. But we don’t give that much attention.’ [Pokhara4].

A participant who had recently finished his degree described what he had learned in school and recognised that people who had not received a formal education were disadvantaged:

‘When I was at school, there was a separate subject for that. In health, there was transmissible disease and how to avoid[…]If people didn’t go to school they have to be made aware by the programmes, or we could do in the radio or the television, broadcasting[…]Government should invest some money for that [*laughing*].’ [Kathmandu5].

Practitioners expressed views on community enthusiasm for programmes and discussed their experiences with running these:

‘Our finding was that they are quite poor in knowledge nowadays and the practice was not so good. No safety and hygiene[…]the attitude was good actually because they really want to learn and adopt the practices, but practice was a mess.’ [Health2].

#### Responsibility for programme administration

When discussing who should take responsibility for directing programmes, many community participants stated that government should do so as the information would be more believable, and more people would attend:

‘Programme initiated by government would be very powerful and effective, rather than from other organisations because people would have more belief, and they would be more supportive if the government can initiate the programme from their own level[…]I think that would be more durable as well, rather than small programmes occasionally from private organisations.’ [Bhaktapur4].

‘I am totally agreed that health-related programmes are pivotal for the community. Local government should conduct such programme in the regular basis. But unfortunately, there is not a single such programme so far.’ [Gulmi5].

However, members of one FGD reported differently:

‘Effectiveness is seen when such programmes are conducted by NGOs as people attend such programmes as compared to the municipalities. People often do not attend the programmes conducted by the municipality. [*Why?*] People are just more interested in programme when the NGOs conduct it.’ [Mustang FGD].

#### Fostering community co-production

Community participants described that, with the exception of female community health volunteers (FCHVs), they have had little to no interaction with stakeholders, and they have no experience of co-production. Healthcare professionals described community members as experts in their own context and that they must be included from the beginning of any initiative to enable effective knowledge sharing and implementation:

‘Community people are the most powerful resources[…]they could be a powerful weapon if they are provided with real information, and they are provided with the capacity to deal with those outbreaks.’ [Health3].

‘They don’t involve the community members, they don’t involve them during the writing phase. So this is what I learned. The community engagement is lacking in the project cycle.’ [Health2].

Inclusion can be facilitated by fostering trust, ensuring that sessions or materials are produced in local languages, and acknowledging customs and beliefs:

‘When we go to communities[…]I engage familiarly with them and I talk simply, let them feel that I am also like them. So they don’t need to be intimidated. I eat with them, drink with them and then they are a little bit friendly, a bit comfortable. When they start to express their feelings then I feel like ok they are now familiar with me[…]after that we start asking them what problems they are having with animals.’ [Livestock3].

‘There’s a language issue, that’s why [*redacted organisation*] uses things like local songs, that’s always quite effective. People like communication in their own language, in their own context.’ [Health4].

‘Once you get the trust, they will listen to you. So how do you get the trust? So one is the language[…]When I speak their language, they talk to me, they have several questions. And once they feel that their question is answered, they sit, and want to know more.’ [Health8].

FCHVs are respected members of communities and responsible for much of the general human and animal health awareness and vaccination programmes that are run in Nepal. FCHVs were mentioned many times by both community and practitioner participants, underlining the potential leverage that these women have:

‘Sometimes FCHVs visit each household for vaccination.’ [Bhaktapur1].

‘Municipality send FCHVs in wards and they spread health-related education to general public.’ [Bhaktapur3].

‘The health post conducts these programmes with the help of female community health workers in different villages. They also provide training and awareness programmes.’ [Pokhara7].

‘We have an extensive network of female community health volunteers. They work in the community, they are not directly affiliated with health institutions. They are often expected to visit every house in the community and they have good relations with the community. We often engage them in the process, so it has been relatively easy for us to work with the community.’ [Health13].

‘In Nepal we have female community health volunteers, so we have to reach to that level, make them aware and make them the leader of the community campaign because they are the ones who are most connected with the villagers, so to prevent misuse of the vaccinations, they will trust these volunteers.’ [Livestock6].

In Gulmi, which was a more rural site than those in Bhaktapur and Pokhara, participants stated that FCHVs began appearing in the community about 12 years ago but these women are more involved in microfinance initiatives than in healthcare, while a participant in the same area talked about the lack of programming:

‘There are FCHVs round here. People talk about conducting programmes but no actions have been taken. After COVID people also have started washing their hands and are taking sanitation seriously but no programmes related to that have been conducted.’ [Gulmi2].

### Conceptual framework

**Fig. 2:**
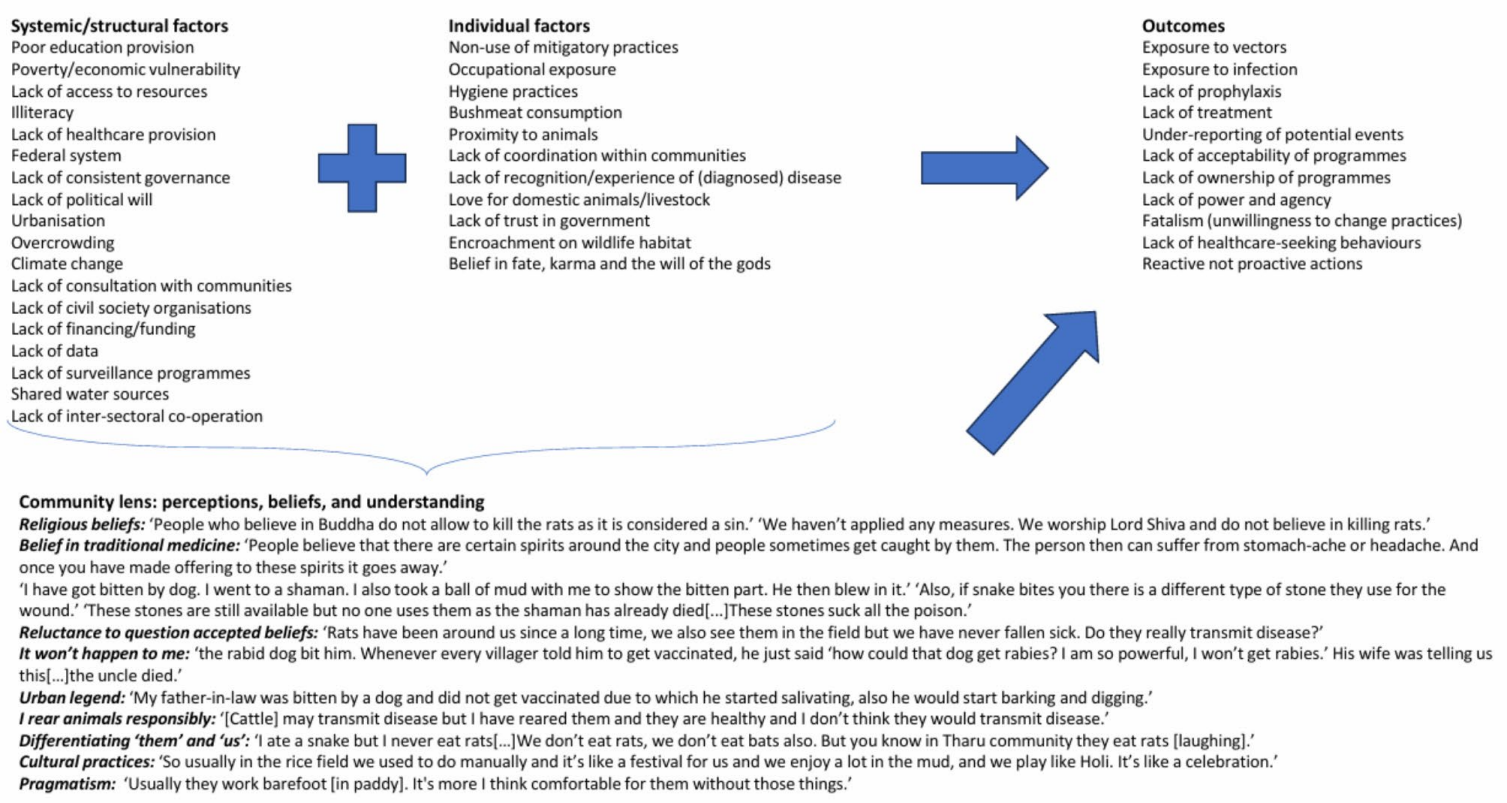
Factors influencing zoonotic disease risk in communities in Nepal

Having spoken to community members, policymakers and healthcare practitioners in the country, and building on the Fundamental Cause Theory [29, 30], which describes the importance of contextualising risk factors, we constructed a conceptual framework incorporating participant views to visualise and describe the many different factors that might influence zoonotic disease risk in Nepal (Fig. 2). This framework has four main sections: the systemic and structural factors, the individual factors, the likely outcomes, and the community context– the lens through which the other factors must be viewed for the outcomes to appear logical and coherent. All quotes in Fig. 2 are from community participants.

## Discussion

In this study, grounded in a critical realist perspective in which context is central, we collaborated with community members to identify aspects of zoonotic and infectious disease that are important to them in their context, in an effort to facilitate future co-production of research and practice around zoonotic disease risks. As far as we are aware, this is the first study to do so in Nepal. There is an increasing realisation by both policymakers and practitioners that working with and within communities is central to effective healthcare initiatives: listening to what community members know, what they feel, what they do (and why), and what they would like to know and do to deal with their own identified priorities [10, 11, 51]. This involves recognising community members as the experts in their own context and taking into consideration language, culture and religion, as exemplified by the participants in this study.

The experience in the informal settlement was especially informative for us because disadvantaged groups lacking power and with limited means of communicating their opinions are potentially more likely to be reluctant to talk if nervous about potential ramifications of doing so, particularly with researchers who look different and where power relations are not clear [52]. Conversely, one of the informal settlement participants took the opportunity to voice her strong views on the political situation in Nepal, and how it was typical that we had taken the time to visit her and ask her opinions, whereas politicians never came to the settlement unless it was to draw up plans to remove it. Participation of marginalised communities or groups may motivate them to think about what kind of changes they would like to make [53]. However, this may be distressing, if people understand that they are unable in practice to make any changes as a result of their situation. Studies have demonstrated that, although communities may be aware of some measures they can take to protect themselves and their communities from potential exposure to disease, they do not do so. This has been seen in studies of behaviour around rabies prevention and vaccination in Pakistan [54], India [55] and Bhutan [56], spillover of bat coronavirus in rural China [57], and spread of Nipah virus in Bangladesh [58], and underlines the centrality of working with communities to understand what might be culturally acceptable and effective, tailoring potential programmes or activities to their specific context. This has been shown in a community-based One Health project in South Africa, where a training programme aimed at increasing disease risk mitigation was specifically designed for local agro-pastoralist communities [59]. This programme, which involved local facilitators who ran workshops with community members, resulted in 98% of participants implementing risk mitigation strategies (e.g., improved animal housing, improved personal hygiene, improved garbage disposal) during a 3-month follow-up period. The authors state that such programmes should be context-dependent, and emphasised the leverage of local facilitators, which encouraged community ownership of the programmes and potential solutions identified by the community members themselves [59].

Key to encouraging co-production of initiatives is recognising that communities are experts and should be consulted at all stages of design and implementation of any initiative. One study examining the feasibility of co-designing a model of care for people with chronic obstructive pulmonary disease in Nepal found that patients were enthusiastic about being involved in designing their own care management plans and the study also successfully engaged healthcare stakeholders. Enablers included the integration of the care plans into the existing health services and involvement of patients and caregivers, while barriers included creation of expectations among patients, the time involved in the co-design process, and involving patients from marginalised communities. However, these barriers might be overcome by engaging community leaders and being explicit about the limitations of the programme from the very beginning of the process [60]. A recent review on engaging communities with communicable disease control in low- and lower-middle-income countries found a range of initiatives that improved effectiveness of communicable disease control programmes: some involved community members in identifying relevant disease control issues, some involved them in developing materials and messages to be used in programmes, and some helped community members form coherent groups (e.g., women’s groups) that then became central to programming [11, 61]. The significance of working in local languages and respecting cultures, relating projects to the everyday life of communities, and ensuring that local leaders such as village heads and FCHVs are involved, was discussed by both community members and practitioners in our study. Leveraging existing experience and structures, such as that provided by FCHVs, may increase receptiveness of communities to messages. This has been demonstrated for diseases such as AIDS and tuberculosis, and for maternal and newborn health in disparate areas of the globe [11]. In a recent paper, Liverani and colleagues discuss the significance of village health volunteers in Laos– these volunteers receive basic healthcare training and are then involved in health promotion, patient referral, and treatment of common and minor illnesses. Local stakeholders interviewed suggested that these volunteers should be fluent in both Lao and the language of their own ethnic group, should be representative of their community, and that more female volunteers should be involved, as many women prefer to discuss health problems with other women [62]. In Nepal, FCHVs are the only link between many rural communities and healthcare facilities and are trusted and respected members of the community [63]. Studies in Nepal demonstrated that involving these FCHVs increased acceptance of contraception, and improved delivery of basic maternity and perinatal care [64–66]. The experience and skills of these volunteers could be leveraged in disease control programmes.

There are obvious disadvantages and limitations to a system that uses FCHVs extensively, including the fact that they are volunteers and so any initiative that depends on them and their goodwill may be precarious [63]. They may also perceive themselves (and be perceived) as inferior to more qualified healthcare workers [67]. Coupled with this is the potential for exploitation, as FCHVs often work alongside, and work similar hours to, salaried health-workers. In 2023, a systematic review of 112 studies from 19 countries found that 59% of unsalaried community healthcare volunteers experienced labour exploitation, and almost one-fifth of these workers had to work for more than 40 h every week to meet their assigned responsibilities [68]. One study examining the motivation of FCHVs in Nepal found that these women, already responsible for household chores and childcare, lacked support from their families, did not feel appreciated by members of their community, and were out-of-pocket as their expenses were not covered. They perceived themselves as disrespected by healthcare workers in the formal sector, and were burdened by bureaucracy that they were ill-equipped to deal with [69]. However, another study found that a feeling of moral duty, the pride that volunteers felt, and the respect gained in their community through their role, helped prevent volunteer attrition [70]. Women in the Nepal study stated that they saw volunteering as an opportunity to make a difference, and they felt pride in their role [69]. It is vital to keep in mind that, as volunteers are almost always female, this imbalance may reinforce accepted gender disparities, and increase inequalities, especially within communities that may be traditional and unaccepting of female empowerment [65, 71].

Working with traditional medicine practitioners, who are based in villages and are often consulted before people visit an allopathic doctor, is another option, especially as studies have shown that some people in Nepal believe that illness is conferred by the gods, and related to karma, fate and destiny, and traditional medicine practitioners are perceived as the only people qualified to alleviate or prevent these illnesses [72, 73]. Another study found that 85% of people living in rural areas of Nepal visit traditional healers before any others, partly as a result of proximity and ease of access, but also as a result of being more culturally accessible than allopathic practitioners may be [74]. In Mozambique, traditional healers have been trained in symptom identification (particularly for infectious diseases such as HIV/AIDS, TB and malaria) and patient referral, and incorporated into the allopathic healthcare system [75–77]. This training resulted in better identification of health issues and an increased number of referrals, although the authors state that clinicians were unwilling to accept these referrals from the healers, as they did not accept their diagnoses [76], and so need to be encouraged to work together with the healers instead of in opposition to them. When healers were trained to perform directly observed therapy for people with HIV, patient adherence to treatment increased, and they reported positive psychosocial effects of the intervention [77]. These findings suggest that integration of traditional medicine practitioners into the allopathic healthcare system, although not straightforward, is achievable if patients, clinicians and healers themselves agree to co-operate. These healers, with an extensive knowledge of local beliefs and what is culturally acceptable, could be involved in designing context-specific strategies to address zoonotic disease risk. Healers could be trained to recognise symptoms of specific diseases, or to at least discuss with community members their behaviours and how this may affect their health. For example, if a person came to the healer after experiencing an animal bite, the healer could refer them to a health post for vaccination, stressing the importance of doing so, or they could even be supplied with injections that they can administer themselves.

Respecting and working with traditional medicine practitioners, who are often consulted before allopathic healthcare representatives, was discussed at the Alma-Ata conference on primary health care in 1978, with the meeting report stating that ‘indigenous practitioners can become important allies in organizing efforts to improve the health of the community’ [78]. Traditional healers are not formally recognised as legitimate health practitioners in Nepal, although their integration into the primary healthcare system in the country is currently being discussed at governmental level [79]. These healers are trusted and respected community members, sharing a culture, and are consulted on emotional, spiritual and psychosocial problems, as well as physical illnesses [79]. Taken together, this suggests that this group of people, who are already present in the community, are a key target for incorporation into public healthcare provision.

A recent review of community-focused responses to the COVID-19 pandemic found that using local languages encouraged marginalised groups to express themselves and participate fully in planning and administering programmes [80]. This was also demonstrated in a scoping review of health system evaluations in conflict-affected countries [81], and in research with highly vulnerable participants in Syria [82]. In our study, participants highlighted the significance of working in local languages, which worked to increase trust between communities and outsiders. We conducted the FGD in Bhaktapur in both Nepali and Newari, as, although all participants were fluent in both languages, some participants felt better able to discuss their views in the language which they identified as their ‘own’, rather than in Nepali. A recent study in Nepal, focused on community knowledge of antimicrobial resistance, found that words relevant to this concept do not exist in Nepali or Awadhi, the language used by participants, and they instead represented these concepts using full sentences and colloquial words, which complicated discussion of the topic [83]. This underlines the importance of understanding the linguistic context in which people live. If able to use their own language when consulting health professionals, participants may use words to explain or understand concepts that they cannot easily communicate in another language, reducing likelihood of misunderstandings and, potentially, misdiagnosis and incorrect treatment [84]. A study on use of local languages to describe women’s health conditions in South Africa found that participants who were able to discuss their health issues in their own indigenous language received more effective treatment more quickly than those who were not [85]. Setting aside practicalities like this, having to communicate with health professionals in a language not usually spoken may reinforce power imbalances that are already present as a result of the health professional potentially being better educated and not from the patient’s locality [84]. Other research demonstrates that maintaining and using indigenous language has a positive effect on health, and can improve quality of care [86, 87].

Related to this is working closely with people who are trusted members of the local community: village heads and community health workers, who are often based in or close to the village they are serving. Peer-led delivery of programmes consistently led to more effective engagement with communities [21], and it is important that community participants perceive the entire research and implementation process as appropriate to their needs, and gives them a space in which to discuss their issues and potential solutions [25]. Trust has been demonstrated as a key factor in other contexts such as the Ebola outbreak in west Africa in 2014 [88]. Involving community members in spreading information on how to protect against the disease resulted in an increased adherence to reducing number of interactions, and observing safe burial practices.

Most practitioners suggested that communities were not consulted before programme implementation, so whether initiatives would be welcome, or even necessary, was unclear. One exception was a veterinarian, who discussed farmers taking part in discussions on what affected their livelihoods, and what they wanted to know about how to prevent their animals becoming ill. This example could be built on, with consultations held with smallholders and farmers, discussing their priorities and explaining what they can do to safeguard both their health and their livelihoods. Interventions that do exist tend to focus on general health, which is an existing platform on which to build co-production initiatives. A scoping review found that implementing community-based interventions through existing platforms (e.g., maternal and antenatal programmes and immunisation campaigns) is effective, reducing prevalence of risky behaviours and reducing infectious disease burden [89]. However, this review did not find a clear answer to whether integrated or stand-alone programming was more effective: stand-alone interventions are easier to implement as they require fewer coordination partners, but integrated interventions that allow delivery of multiple vaccinations or treatments may be cheaper [89].

Participants claimed interest in learning more about zoonotic disease and taking part in health programmes, especially if they were tailored to prevalent issues in their local community. When discussing who should take responsibility for directing programmes, many participants stated that government should do so as the information would be more believable, and more people would attend. Interestingly, one systematic review found that programmes run jointly by different stakeholders, e.g., NGOs and government representatives, were more sustainable than others, probably as a result of political support and concomitant financing [90]. A study focused on messaging around Ebola during the 2014 outbreak in Liberia found that, as the outbreak worsened and more people were affected, government messages, originally thought to be ‘false’, became more influential and people began to be more engaged [91]. These findings suggest that, as discussed by some participants, government support may be central to an effective intervention. Feedback to communities, potentially presenting findings on presence of disease or success of existing initiatives, was mentioned as important. To encourage participation, community members should be shown a ‘result’ of their time and effort in attending programmes or providing researchers with information.

Women are more likely to be the main caregivers for livestock, and for children, as they are often at home during the day, and women-led groups may effectively support community initiatives, although there needs to be a specific benefit in participating, as these initiatives would involve more (and likely unpaid) work. Mustang participants discussed the women’s group in their local community, who have taken on the responsibility for addressing issues around community dogs. These types of groups might be a natural forum for discussing zoonotic disease and potential mitigation of both risk and spread. Working with FCHVs, context-specific initiatives, run by and for the community, that do not rely on government-led programmes, could be designed and implemented. These types of women-led groups have been demonstrated to be effective in areas as different as agricultural development in west Bengal, India [92] and improving perinatal care [64] and female household agency in Nepal itself [93].

**Fig. 3:**
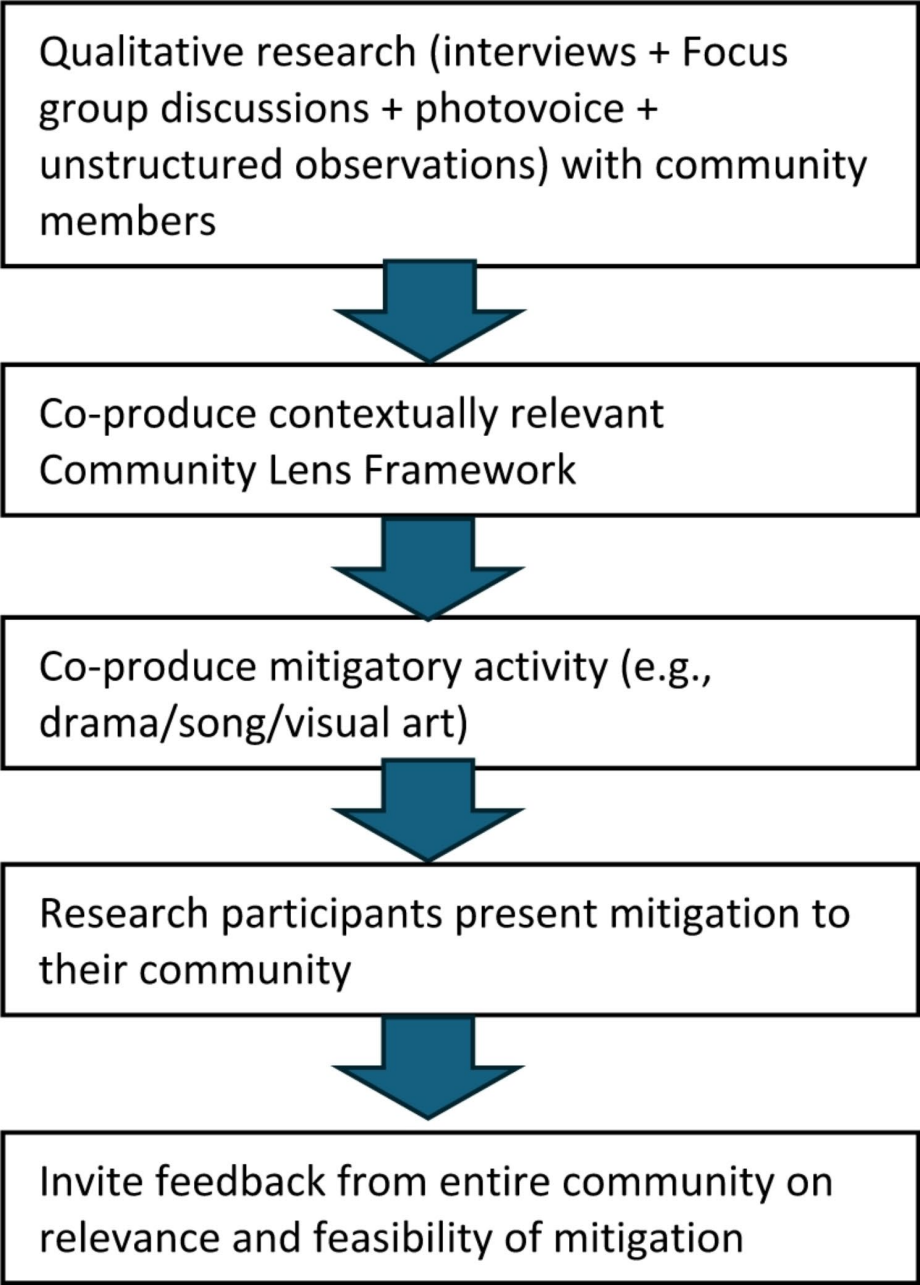
Design of a research project involving co-production of a mitigatory activity

Through discussions with community members, healthcare practitioners and policymakers, attempting to clarify their priorities, our research demonstrated the importance of these individual and structural factors, but also supports the centrality of trying to see through a ‘community lens’, involving less concrete, more subtle factors such as perceptions, beliefs and understandings of the community in which the research or intervention is situated. Barnett and colleagues state that epidemiological research around zoonotic disease tends to be didactic and focused on behaviour change, which places the focus centrally on the population in question, and so interventions informed by this are unlikely to be effective as they ignore social, cultural and economic factors. This insight supports the conceptual framework, in that it underlines the need to have a more holistic understanding [94]. For example, living with rodents or other ubiquitous pest animals, drinking polluted water as it is all that is accessible, eating bushmeat as it is freely available, are examples of factors that some communities experience on a daily basis, and need to be considered when we look at WHY people are at risk. This is why a One Health approach to this kind of research is so important: we need to take into account all aspects of health, related to animals, humans and the environment, in an attempt to create a holistic solution to issues that are identified by the community, rather than simply focusing on human health. This will allow the design of mitigatory interventions that take into account issues around the inability to avoid certain risk factors, without which these interventions are unlikely to be effective. We present a potential design for a research project that incorporates the community at all steps of a mitigatory activity in Fig. 3.

However, awareness of availability or otherwise of resources to channel into research and implementation must also be factored into any initiative. As Agyepong and colleagues note, a country has to have secure human and financial resources before it can strengthen capacity for co-production of health research and programming [95]. One participant in our study pointed out that these structural issues will affect community responses, stating that people might attend programmes but are not necessarily able to follow advice. If the closest health post is 4 hours’ walk away, and there is no pharmacy to fill a prescription, then why should people spend time and energy attending programmes? Clarity around how effective an intervention might be is essential to manage community expectations, as interventions and research alone, without structural change, are unlikely to have a significant effect on other aspects of community life, such as poverty and lack of access to resources [71]. The success of these initiatives should be measured by communities themselves, in sessions convened for people to give their feedback: did communities find initiatives useful, what worked, what did not work, and why not.

### Limitations

Limitations include that we focused on six regions of Nepal, and participants in other areas may have had different experiences. Many interviews involved translation: concepts in Nepali may differ from those in English, with different nuances and analogies that may not fully make sense in another language. Most community participants had not been interviewed before and so we worked to make them as comfortable as possible with the process. ADB is a different demographic to the participants, which may have affected perceived power relations. She worked hard to build a rapport with participants, explaining what we were doing and why, and discussing her experience living and working in Nepal, which is recommended as a method of encouraging participants to share their experiences [96]. During planning, interviewing, analysis and writing stages of the study she assessed her positionality, assumptions, preconceptions, values and motivations for doing the research. With these caveats, we believe this study contributes to the limited body of evidence on potential co-production of public health and infectious disease programming in Nepal.

## Conclusions

The emergence of diseases such as Ebola virus disease and COVID-19 have underlined the importance of using an inclusive One Health lens to holistically address issues around human, animal and environmental health, and ensuring that communities are included in any attempt to understand drivers behind the emergence of these zoonotic diseases. This study demonstrated the importance of working with communities to understand context and priorities, and that critical realism adds meaning in this type of exploratory study. Using indigenous languages, respecting community cultures, listening to viewpoints, involving community leadership (religious leaders, traditional medicine practitioners, village heads, community health workers), and working with or through existing programmes are all factors that could make these more effective. Engagement between health professionals and communities, tailoring interventions to work with local priorities and co-developing effective solutions addressing drivers of zoonotic disease, are positive steps toward achieving workable solutions to potential disease risk.

## Data Availability

All relevant data generated or analysed during this study are included in this published paper.

## Abbreviations

FCHV: Female community health volunteer
FGD: Focus group discussion

## References

[1] Holmes EC. COVID-19 - lessons for zoonotic disease. Science. 2022;375(6585):1114–5. 35271309 10.1126/science.abn2222

[2] Karesh WB, Dobson A, Lloyd-Smith JO, Lubroth J, Dixon MA, Bennett M, et al. Ecology of zoonoses: natural and unnatural histories. Lancet. 2012;380(9857):1936–45. 23200502 10.1016/S0140-6736(12)61678-X PMC7138068

[3] Taylor LH, Latham SM, Woolhouse ME. Risk factors for human disease emergence. Philos Trans R Soc Lond B Biol Sci. 2001;356(1411):983–9. 11516376 10.1098/rstb.2001.0888 PMC1088493

[4] Adisasmito WB, Almuhairi S, Barton Behravesh C, Bilivogui P, Bukachi SA, Casas N, et al. One health action for health security and equity. Lancet. 2023;401(10376):530–3. 36682373 10.1016/S0140-6736(23)00086-7 PMC9851626

[5] Ashworth HC, Roux TL, Buggy CJ. Healthcare accessibility in the rural plains (terai) of Nepal: physical factors and associated attitudes of the local population. Int Health. 2019;11(6):528–35. 30916330 10.1093/inthealth/ihz008

[6] Asaaga FA, Young JC, Srinivas PN, Seshadri T, Oommen MA, Rahman M, et al. Co-production of knowledge as part of a onehealth approach to better control zoonotic diseases. PLOS Global Public Health. 2022;2(3):e0000075. 36962247 10.1371/journal.pgph.0000075 PMC10021618

[7] MonkeyFeverRisk. What is KFD? 2024 [Available from: https://monkeyfeverrisk.ceh.ac.uk/what-kfd

[8] World Bank. World Bank Country and Lending Groups. 2023 [Available from: https://datahelpdesk.worldbank.org/knowledgebase/articles/906519-world-bank-country-and-lending-groups

[9] World Bank. Current health expenditure (% of GDP) - Nepal. 2020 [Available from: https://data.worldbank.org/indicator/SH.XPD.CHEX.GD.ZS?locations=NP

[10] Osborne J, Paget J, Napier D, Giles-Vernick T, Kutalek R, Rodyna R, et al. Addressing vulnerabilities in communities facing infectious disease threats: a need for social science-driven assessments. J Glob Health. 2021;11:03003. 33604031 10.7189/jogh.11.03003 PMC7872718

[11] Questa K, Das M, King R, Everitt M, Rassi C, Cartwright C, et al. Community engagement interventions for communicable disease control in low- and lower- middle-income countries: evidence from a review of systematic reviews. Int J Equity Health. 2020;19(1):51. 32252778 10.1186/s12939-020-01169-5 PMC7137248

[12] Tugendhaft A, Hofman K, Danis M, Kahn K, Erzse A, Twine R, et al. Deliberative engagement methods on health care priority-setting in a rural South African community. Health Policy Plann. 2021;36(8):1279–91. 10.1093/heapol/czab005 PMC8428615 34051093

[13] Turk E, Durrance-Bagale A, Han E, Bell S, Rajan S, Lota MMM, et al. International experiences with co-production and people centredness offer lessons for covid-19 responses. BMJ. 2021;372:m4752. 33593813 10.1136/bmj.m4752 PMC7879267

[14] Farr M, Davies P, Andrews H, Bagnall D, Brangan E, Davies R. Co-producing knowledge in health and social care research: reflections on the challenges and ways to enable more equal relationships. Humanit Social Sci Commun. 2021;8(1):105.

[15] Tembo D, Hickey G, Montenegro C, Chandler D, Nelson E, Porter K, et al. Effective engagement and involvement with community stakeholders in the co-production of global health research. BMJ. 2021;372:n178. 33593805 10.1136/bmj.n178 PMC7879275

[16] USAID. Bringing the community together to plan for disease outbreaks and other emergencies.; 2019.

[17] Adhikari B, Pell C, Cheah PY. Community engagement and ethical global health research. Global Bioeth. 2020;31(1):1–12. 10.1080/11287462.2019.1703504 PMC6968663 32002019

[18] Wilkinson A, Parker M, Martineau F, Leach M. Engaging ‘communities’: anthropological insights from the West African Ebola epidemic. Philos Trans R Soc Lond B Biol Sci. 2017;372(1721). 10.1098/rstb.2016.0305 PMC5394643 28396476

[19] Kutalek R, Wang S, Fallah M, Wesseh CS, Gilbert J. Ebola interventions: listen to communities. Lancet Glob Health. 2015;3(3):e131. 25618243 10.1016/S2214-109X(15)70010-0

[20] Wolking D, Karmacharya D, Bista M, Shrestha R, Pandit P, Sharma A, et al. Vulnerabilities for exposure to emerging infectious disease at urban settlements in Nepal. EcoHealth. 2020;17(3):345–58. 33206274 10.1007/s10393-020-01499-4 PMC7672689

[21] Brunton G, Thomas J, O’Mara-Eves A, Jamal F, Oliver S, Kavanagh J. Narratives of community engagement: a systematic review-derived conceptual framework for public health interventions. BMC Public Health. 2017;17(1):944. 29228932 10.1186/s12889-017-4958-4 PMC5725895

[22] Centers for Disease Control and Prevention. Principles of community engagement. 1997. Available from: https://www.atsdr.cdc.gov/communityengagement/pce_intro.html

[23] Vennik FD, van de Bovenkamp HM, Putters K, Grit KJ. Co-production in healthcare: rhetoric and practice. Int Rev Admin Sci. 2016;82(1):150–68.

[24] Popay J. Community empowerment and health improvement: the english experience. In: Morgan A, Davies M, Ziglio E, editors. Health Assets in a Global Context: Theory, Methods, Action. New York, NY.: Springer New York.; 2010.

[25] Yoeli H, Dhital R, Hermaszewska S, Sin J. A meta-ethnography of participatory health research and co-production in Nepal. Soc Sci Med. 2022;301:114955. 35452892 10.1016/j.socscimed.2022.114955

[26] Li HY, Zhu GJ, Zhang YZ, Zhang LB, Hagan EA, Martinez S, et al. A qualitative study of zoonotic risk factors among rural communities in Southern China. Int Health. 2020;12(2):77–85. 32040190 10.1093/inthealth/ihaa001 PMC7017878

[27] Jacobsen KH, Aguirre AA, Bailey CL, Baranova AV, Crooks AT, Croitoru A, et al. Lessons from the Ebola outbreak: action items for emerging infectious disease preparedness and response. EcoHealth. 2016;13(1):200–12. 26915507 10.1007/s10393-016-1100-5 PMC7087787

[28] Durrance-Bagale A, Rudge JW, Singh NB, Belmain SR, Howard N. Drivers of zoonotic disease risk in the Indian Subcontinent: a scoping review. One Health. 2021;13:100310. 34458546 10.1016/j.onehlt.2021.100310 PMC8379342

[29] Link BG, Phelan J. Social conditions as fundamental causes of disease. J Health Soc Behav. 1995;Spec No:80–94. 7560851

[30] Phelan JC, Link BG, Tehranifar P. Social conditions as fundamental causes of health inequalities: theory, evidence, and policy implications. J Health Soc Behav. 2010;51(Suppl):S28–40. 20943581 10.1177/0022146510383498

[31] Noppert GA, Hegde ST, Kubale JT. Exposure, susceptibility, and recovery: a framework for examining the intersection of the social and physical environments and infectious disease risk. Am J Epidemiol. 2023;192(3):475–82. 36255177 10.1093/aje/kwac186 PMC10372867

[32] Hewlett BS. Evolutionary cultural anthropology: containing Ebola outbreaks and explaining Hunter-Gatherer childhoods. Curr Anthropol. 2016;57(S13):S27–37.

[33] Haigh F, Kemp L, Bazeley P, Haigh N. Developing a critical realist informed framework to explain how the human rights and social determinants of health relationship works. BMC Public Health. 2019;19(1):1571. 31775689 10.1186/s12889-019-7760-7 PMC6882063

[34] Fletcher AJ. Applying critical realism in qualitative research: methodology meets method. Int J Soc Res Methodol. 2017;20(2):181–94.

[35] Greenhalgh J, Manzano A. Understanding ‘context’ in realist evaluation and synthesis. Int J Soc Res Methodol. 2022;25(5):583–95.

[36] Dore I. Doing knowing ethically– where social work values meet critical realism. Ethics Social Welf. 2019;13(4):377–91.

[37] Palinkas LA, Horwitz SM, Green CA, Wisdom JP, Duan N, Hoagwood K. Purposeful sampling for qualitative data collection and analysis in mixed method implementation research. Adm Policy Ment Health. 2015;42(5):533–44. 24193818 10.1007/s10488-013-0528-y PMC4012002

[38] Patton M. Qualitative research & evaluation methods: integrating theory and practice. Sage; 2014.

[39] Maxwell J. A realist approach for qualitative research. LA, USA: Sage; 2012.

[40] Fetters MD, Rubinstein EB. The 3 Cs of content, context, and concepts: a practical approach to recording unstructured field observations. Ann Fam Med. 2019;17(6):554–60. 31712294 10.1370/afm.2453 PMC6846267

[41] Geertz C. Thick description: toward an interpretive theory of culture. Interpretation of cultures: selected essays. New York, NY, USA.: Basic Books; 2000.

[42] Braun V, Clarke V. Thematic analysis: a practical guide. SAGE Publications Ltd; 2021.

[43] Byrne D. A worked example of Braun and Clarke’s approach to reflexive thematic analysis. Qual Quant. 2022;56(3):1391–412.

[44] Wiltshire G, Ronkainen N. A realist approach to thematic analysis: making sense of qualitative data through experiential, inferential and dispositional themes. J Crit Realism. 2021;20(2):159–80.

[45] Fryer T. A critical realist approach to thematic analysis: producing causal explanations. J Crit Realism. 2022;21(4):365–84.

[46] Dwyer SC, Buckle JL. The space between: on being an insider-outsider in qualitative research. Int J Qualitative Methods. 2009;8(1):54–63.

[47] Green J, Thorogood N. Qualitative methods for health research, 3rd Edition.: SAGE Publications; 2014.

[48] Sims D. Reimaging reflexivity through a critical theoretical framework: autoethnographic narratives on becoming a (de)colonised researcher. Crit Stud Teach Learn (CriSTaL). 2023;11(1).

[49] Berger R. Now I see it, now I don’t: researcher’s position and reflexivity in qualitative research. Qualitative Res. 2015;15(2):219–34.

[50] Abimbola S. The foreign gaze: authorship in academic global health. BMJ Glob Health. 2019;4(5):e002068. 31750005 10.1136/bmjgh-2019-002068 PMC6830280

[51] Mwatondo A, Rahman-Shepherd A, Hollmann L, Chiossi S, Maina J, Kurup KK, et al. A global analysis of one health networks and the proliferation of one health collaborations. Lancet. 2023;401(10376):605–16. 36682370 10.1016/S0140-6736(22)01596-3

[52] Adler P, Adler P. The reluctant respondent. In: Holstein JA, JF. G, editors. Inside interviewing: new lenses. new concerns,: SAGE Publications Inc; 2003.

[53] Nykiforuk CIJ, Vallianatos H, Nieuwendyk LM. Photovoice as a method for revealing community perceptions of the built and social environment. Int J Qualitative Methods. 2011;10(2):103–24. 10.1177/160940691101000201 PMC4933584 27390573

[54] Khan A, Ayaz R, Mehtab A, Naz K, Haider W, Gondal MA, et al. Knowledge, attitude & practices (KAPs) regarding rabies endemicity among the community members, Pakistan. Acta Trop. 2019;200:105156. 31491398 10.1016/j.actatropica.2019.105156

[55] Brookes VJ, Gill GS, Singh BB, Sandhu BS, Dhand NK, Aulakh RS, et al. Challenges to human rabies elimination highlighted following a rabies outbreak in bovines and a human in Punjab, India. Zoonoses Public Health. 2019;66(3):325–36. 30779303 10.1111/zph.12568

[56] Rinchen S, Tenzin T, Hall D, van der Meer F, Sharma B, Dukpa K, et al. A community-based knowledge, attitude, and practice survey on rabies among cattle owners in selected areas of Bhutan. PLoS Negl Trop Dis. 2019;13(4):e0007305. 30933984 10.1371/journal.pntd.0007305 PMC6459539

[57] Li H, Mendelsohn E, Zong C, Zhang W, Hagan E, Wang N, et al. Human-animal interactions and bat coronavirus spillover potential among rural residents in Southern China. Biosaf Health. 2019;1(2):84–90. 32501444 10.1016/j.bsheal.2019.10.004 PMC7148670

[58] Nahar N, Paul RC, Sultana R, Gurley ES, Garcia F, Abedin J, et al. Raw Sap consumption habits and its association with knowledge of Nipah virus in two endemic districts in Bangladesh. PLoS ONE. 2015;10(11):e0142292. 26551202 10.1371/journal.pone.0142292 PMC4638332

[59] Berrian AM, Smith MH, van Rooyen J, Martínez-López B, Plank MN, Smith WA, et al. A community-based one health education program for disease risk mitigation at the human-animal interface. One Health. 2018;5:9–20. 29270459 10.1016/j.onehlt.2017.11.002 PMC5734692

[60] Yadav UN, Lloyd J, Baral KP, Bhatta N, Mehta S, Harris MF. Using a co-design process to develop an integrated model of care for delivering self-management intervention to multi-morbid COPD people in rural Nepal. Health Res Policy Syst. 2021;19(1):17. 33568139 10.1186/s12961-020-00664-z PMC7874656

[61] Prost A, Colbourn T, Seward N, Azad K, Coomarasamy A, Copas A, et al. Women’s groups practising participatory learning and action to improve maternal and newborn health in low-resource settings: a systematic review and meta-analysis. Lancet. 2013;381(9879):1736–46. 23683640 10.1016/S0140-6736(13)60685-6 PMC3797417

[62] Liverani M, Phongluxa K, Phommasone K, Chew R, Chandna A, Pongvongsa T, et al. Prospects for the development of community-based care in remote rural areas: a stakeholder analysis in Laos. BMC Health Serv Res. 2024;24(1):55. 38212788 10.1186/s12913-023-10523-6 PMC10782664

[63] Khatri RB, Mishra SR, Khanal V. Female community health volunteers in community-based health programs of Nepal: future perspective. Front Public Health. 2017;5. 10.3389/fpubh.2017.00181 PMC5519587 28785555

[64] Morrison J, Tamang S, Mesko N, Osrin D, Shrestha B, Manandhar M, et al. Women’s health groups to improve perinatal care in rural Nepal. BMC Pregnancy Childbirth. 2005;5(1):6. 15771772 10.1186/1471-2393-5-6 PMC1079874

[65] Panday S, Bissell P, van Teijlingen E, Simkhada P. The contribution of female community health volunteers (FCHVs) to maternity care in Nepal: a qualitative study. BMC Health Serv Res. 2017;17(1):623. 28870185 10.1186/s12913-017-2567-7 PMC5584032

[66] Shrestha S. Increasing contraceptive acceptance through empowerment of female community health volunteers in rural Nepal. J Health Popul Nutr. 2002;20(2):156–65. 12186196

[67] Schwarz D, Sharma R, Bashyal C, Schwarz R, Baruwal A, Karelas G, et al. Strengthening Nepal’s female community health volunteer network: a qualitative study of experiences at two years. BMC Health Serv Res. 2014;14(1):473. 25301105 10.1186/1472-6963-14-473 PMC4282192

[68] Ballard M, Olaniran A, Iberico MM, Rogers A, Thapa A, Cook J, et al. Labour conditions in dual-cadre community health worker programmes: a systematic review. Lancet Glob Health. 2023;11(10):e1598–608. 37734803 10.1016/S2214-109X(23)00357-1

[69] Panday S, Teijlingen EV, Barnes A. Exploring the motivations of female community health volunteers in primary healthcare provision in rural Nepal: a qualitative study. PLOS Glob Public Health. 2024;4(8):e0003428. 39088488 10.1371/journal.pgph.0003428 PMC11293747

[70] Glenton C, Scheel IB, Pradhan S, Lewin S, Hodgins S, Shrestha V. The female community health volunteer programme in Nepal: decision makers’ perceptions of volunteerism, payment and other incentives. Soc Sci Med. 2010;70(12):1920–7. 20382464 10.1016/j.socscimed.2010.02.034

[71] Scobie K, Lambin X, Telfer S, Rasahivelo MF, Raheliarison RN, Rajerison M, et al. Living with rodent pests: unifying stakeholder interests to prioritise pest management in rural Madagascar. People Nat. 2023;5(2):713–25.

[72] Marahatta SB, Yadav RK, Giri D, Lama S, Rijal KR, Mishra SR, et al. Barriers in the access, diagnosis and treatment completion for tuberculosis patients in central and Western Nepal: a qualitative study among patients, community members and health care workers. PLoS ONE. 2020;15(1):e0227293. 31940375 10.1371/journal.pone.0227293 PMC6961875

[73] Paudel M, Javanparast S, Dasvarma G, Newman L. Religio-cultural factors contributing to perinatal mortality and morbidity in mountain villages of Nepal: implications for future healthcare provision. PLoS ONE. 2018;13(3):e0194328. 29544226 10.1371/journal.pone.0194328 PMC5854484

[74] Pham TV, Koirala R, Kohrt BA. Satisfaction in the soul: common factors theory applied to traditional healers in rural Nepal. Ethos. 2020;48(1):93–128. 33012879 10.1111/etho.12263 PMC7531438

[75] Audet CM, Salato J, Blevins M, Amsalem D, Vermund SH, Gaspar F. Educational intervention increased referrals to allopathic care by traditional healers in three high HIV-prevalence rural districts in Mozambique. PLoS ONE. 2013;8(8):e70326. 23936407 10.1371/journal.pone.0070326 PMC3731350

[76] Audet CM, Hamilton E, Hughart L, Salato J. Engagement of traditional healers and birth attendants as a controversial proposal to extend the HIV health workforce. Curr HIV/AIDS Rep. 2015;12(2):238–45. 25855337 10.1007/s11904-015-0258-8 PMC4430841

[77] Audet CM, Pettapiece-Phillips M, Tian Y, Shepherd BE, Vermund SH, Salato J. If it weren’t for my traditional healer, I would be dead: engaging traditional healers to support people living with HIV in rural Mozambique. PLoS ONE. 2022;17(6):e0270565. 35763519 10.1371/journal.pone.0270565 PMC9239464

[78] United Nations, World Health Organization. editors. International Conference on Primary Health Care. Alma-Ata, USSR, 6–12 September 1978.

[79] Subedi B. Perspective chapter: integrating traditional healers into the National Health Care System– a review and reflection. In: Christian R, editor. Rural health. Rijeka: IntechOpen; 2023. Ch. 2.

[80] Loewenson R, Colvin CJ, Szabzon F, Das S, Khanna R, Coelho VSP, et al. Beyond command and control: a rapid review of meaningful community-engaged responses to COVID-19. Glob Public Health. 2021;16(8–9):1439–53. 33734007 10.1080/17441692.2021.1900316

[81] Marzouk M, Durrance-Bagale A, Lam ST, Nagashima-Hayashi M, Ung M, Aribou ZM, et al. Health system evaluation in conflict-affected countries: a scoping review of approaches and methods. Confl Health. 2023;17(1):30. 37337225 10.1186/s13031-023-00526-9 PMC10280875

[82] Douedari Y, Alhaffar M, Duclos D, Al-Twaish M, Jabbour S, Howard N. We need someone to deliver our voices’: reflections from conducting remote qualitative research in Syria. Confl Health. 2021;15(1):28. 33865454 10.1186/s13031-021-00361-w PMC8052531

[83] Parajuli A, Garbovan L, Bhattarai B, Arjyal A, Baral S, Cooke P, et al. Exploring community insights on antimicrobial resistance in Nepal: a formative qualitative study. BMC Health Serv Res. 2024;24(1):57. 38212733 10.1186/s12913-023-10470-2 PMC10782613

[84] Al Shamsi H, Almutairi AG, Al Mashrafi S, Al Kalbani T. Implications of language barriers for healthcare: a systematic review. Oman Med J. 2020;35(2):e122. 32411417 10.5001/omj.2020.40 PMC7201401

[85] Rasweswe MM, Peu MD, Mulaudzi FM. The importance of local language in healthcare: naming and defining dysmenorrhea. J Communication Healthc. 2023;16(2):205–14. 10.1080/17538068.2022.2094690 37401880

[86] Whalen D, Moss M, Baldwin D. Healing through language: positive physical health effects of indigenous language use [version 1; peer review: 2 approved with reservations]. F1000Research. 2016;5(852).

[87] Hemberg J, Sved E. The significance of communication and care in one’s mother tongue: patients’ views. Nordic J Nurs Res. 2021;41(1):42–53.

[88] Mbonye AK, Wamala JF, Nanyunja M, Opio A, Makumbi I, Aceng JR. Ebola viral hemorrhagic disease outbreak in West Africa- lessons from Uganda. Afr Health Sci. 2014;14(3):495–501. 25352864 10.4314/ahs.v14i3.1 PMC4209631

[89] Bhutta ZA, Salam RA, Das JK, Lassi ZS. Tackling the existing burden of infectious diseases in the developing world: existing gaps and the way forward. Infect Dis Poverty. 2014;3(1):28. 25105015 10.1186/2049-9957-3-28 PMC4124963

[90] Shigayeva A, Coker RJ. Communicable disease control programmes and health systems: an analytical approach to sustainability. Health Policy Plan. 2015;30(3):368–85. 24561988 10.1093/heapol/czu005

[91] Abramowitz S, McKune SL, Fallah M, Monger J, Tehoungue K, Omidian PA. The opposite of denial: social learning at the onset of the Ebola emergency in Liberia. J Health Communication. 2017;22(sup1):59–65. 28854129 10.1080/10810730.2016.1209599

[92] Carter L, Cosijn M, Williams LJ, Chakraborty A, Kar S. Including marginalised voices in agricultural development processes using an ethical community engagement framework in West Bengal, India. Sustain Sci. 2022;17(2):485–96.

[93] Gram L, Skordis-Worrall J, Manandhar DS, Strachan D, Morrison J, Saville N, et al. The long-term impact of community mobilisation through participatory women’s groups on women’s agency in the household: a follow-up study to the Makwanpur trial. PLoS ONE. 2018;13(5):e0197426. 29758071 10.1371/journal.pone.0197426 PMC5951552

[94] Barnett T, Pfeiffer DU, Ahasanul Hoque M, Giasuddin M, Flora MS, Biswas PK, et al. Practising co-production and interdisciplinarity: challenges and implications for one health research. Prev Vet Med. 2020;177:104949. 32203814 10.1016/j.prevetmed.2020.104949 PMC7218707

[95] Agyepong IA, Godt S, Sombie I, Binka C, Okine V, Ingabire M-G. Strengthening capacities and resource allocation for co-production of health research in low and middle income countries. BMJ. 2021;372:n166. 33593725 10.1136/bmj.n166 PMC7879269

[96] Gilmore B. Realist evaluations in low- and middle-income countries: reflections and recommendations from the experiences of a foreign researcher. BMJ Global Health. 2019;4(5):e001638. 31749993 10.1136/bmjgh-2019-001638 PMC6830045

